# Non-Invasive Prenatal Chromosomal Aneuploidy Testing - Clinical Experience: 100,000 Clinical Samples

**DOI:** 10.1371/journal.pone.0109173

**Published:** 2014-10-07

**Authors:** Ron M. McCullough, Eyad A. Almasri, Xiaojun Guan, Jennifer A. Geis, Susan C. Hicks, Amin R. Mazloom, Cosmin Deciu, Paul Oeth, Allan T. Bombard, Bill Paxton, Nilesh Dharajiya, Juan-Sebastian Saldivar

**Affiliations:** Clinical Science, Sequenom Laboratories, San Diego, California, United States of America; Emory University School Of Medicine, United States of America

## Abstract

**Objective:**

As the first laboratory to offer massively parallel sequencing-based noninvasive prenatal testing (NIPT) for fetal aneuploidies, Sequenom Laboratories has been able to collect the largest clinical population experience data to date, including >100,000 clinical samples from all 50 U.S. states and 13 other countries. The objective of this study is to give a robust clinical picture of the current laboratory performance of the MaterniT21 PLUS LDT.

**Study Design:**

The study includes plasma samples collected from patients with high-risk pregnancies in our CLIA–licensed, CAP-accredited laboratory between August 2012 to June 2013. Samples were assessed for trisomies 13, 18, 21 and for the presence of chromosome Y-specific DNA. Sample data and ad hoc outcome information provided by the clinician was compiled and reviewed to determine the characteristics of this patient population, as well as estimate the assay performance in a clinical setting.

**Results:**

NIPT patients most commonly undergo testing at an average of 15 weeks, 3 days gestation; and average 35.1 years of age. The average turnaround time is 4.54 business days and an overall 1.3% not reportable rate. The positivity rate for Trisomy 21 was 1.51%, followed by 0.45% and 0.21% rate for Trisomies 18 and 13, respectively. NIPT positivity rates are similar to previous large clinical studies of aneuploidy in women of maternal age ≥35 undergoing amniocentesis. In this population 3519 patients had multifetal gestations (3.5%) with 2.61% yielding a positive NIPT result.

**Conclusion:**

NIPT has been commercially offered for just over 2 years and the clinical use by patients and clinicians has increased significantly. The risks associated with invasive testing have been substantially reduced by providing another assessment of aneuploidy status in high-risk patients. The accuracy and NIPT assay positivity rate are as predicted by clinical validations and the test demonstrates improvement in the current standard of care.

## Introduction

Since the first commercial offering of Non-invasive Prenatal Testing (NIPT) by massively parallel sequencing (MPS) in October of 2011, the clinical adoption of NIPT for use in the screening of high risk pregnant patients for the detection of chromosome aneuploidies has grown significantly. The high sensitivity and specificity from multiple clinical validations [1]–[6], the non-invasive aspect of the testing, and the endorsement of key organizations including the American Congress of Obstetrics and Gynecology (ACOG), the Society of Maternal and Fetal Medicine (SMFM) [7], the National Society of Genetic Counselors (NSGC) [8] and, the International Society of Prenatal Diagnosis (ISPD) [9] has resulted in many institutions adopting NIPT within the scope of the standard of care for aneuploidy testing in high risk pregnancies.

As the first laboratory to offer massively parallel sequencing based NIPT for aneuploidy testing, we have been able to accrue the largest clinical experience population dataset to-date including samples from all 50 U.S. states, as well as 13 international countries. From these 100,000 clinical samples we are able to accurately convey the clinical laboratory experience for healthcare providers who select NIPT, examine the results of testing as compared to previous clinical validations and current standards of care, assess the clinical impact on a broader scale, and include examples of the unique clinical findings that were uncovered through testing. The objective of this study is to give a complete and robust clinical picture of the current performance of NIPT for trisomy 13, 18, and 21.

## Methods

All data contained within this study were generated in our CLIA and CAP accredited laboratory from August 2012 to June 2013. Samples were tested for trisomy 13, 18 and 21 as well as presence or absence of Chromosome Y. Samples from high risk pregnancies as defined by advanced maternal age, family or personal history, ultrasound abnormalities, or positive serum screening with gestational age 10 weeks' or greater as determined by last menstrual period (LMP) or ultrasound were accessioned into the laboratory and included in this analysis. Testing was performed on whole blood samples collected in either EDTA or cell-free DNA BCT tubes (Streck Inc.; Omaha, NE) or on processed plasma that was shipped and received frozen. Circulating cell free DNA (ccfDNA) was extracted from plasma using QiAmp circulating nucleic acid kit (Qiagen; Valencia, CA), converted into indexed sequencing libraries multiplexed, clustered, and sequenced on the HiSeq2000 (Illumina, Inc.; San Diego, CA) [3].

All samples were reviewed by a laboratory director prior to the final reporting of results to the ordering physician. Samples with insufficient fetal DNA were classified as quantity not sufficient using a previously described method [24]. Samples failing all other laboratory quality metric including library and sequencing passing criteria were classified as other not reportable etiologies. The anonymized data analyzed for this retrospective study was obtained from existing patient data all of whom signed informed consent prior to testing. All patient data that was generated as a result of the MaterniT21 Plus LDT assay was de-identified and combined for analysis in compliance with FDA Guidance Document “Informed Consent for In Vitro Diagnostic Device Studies Using Leftover Human Specimens that are Not Individually Identifiable” issued on April 25, 2006 and is exempt from IRB review. We are reporting on the overall clinical experience with the assay (positivity rates, not reportable rates, redraw success rates, etc.), and do not provide detailed descriptions of the individual cases.

## Results

### The Laboratory Experience

**Figure 1:**
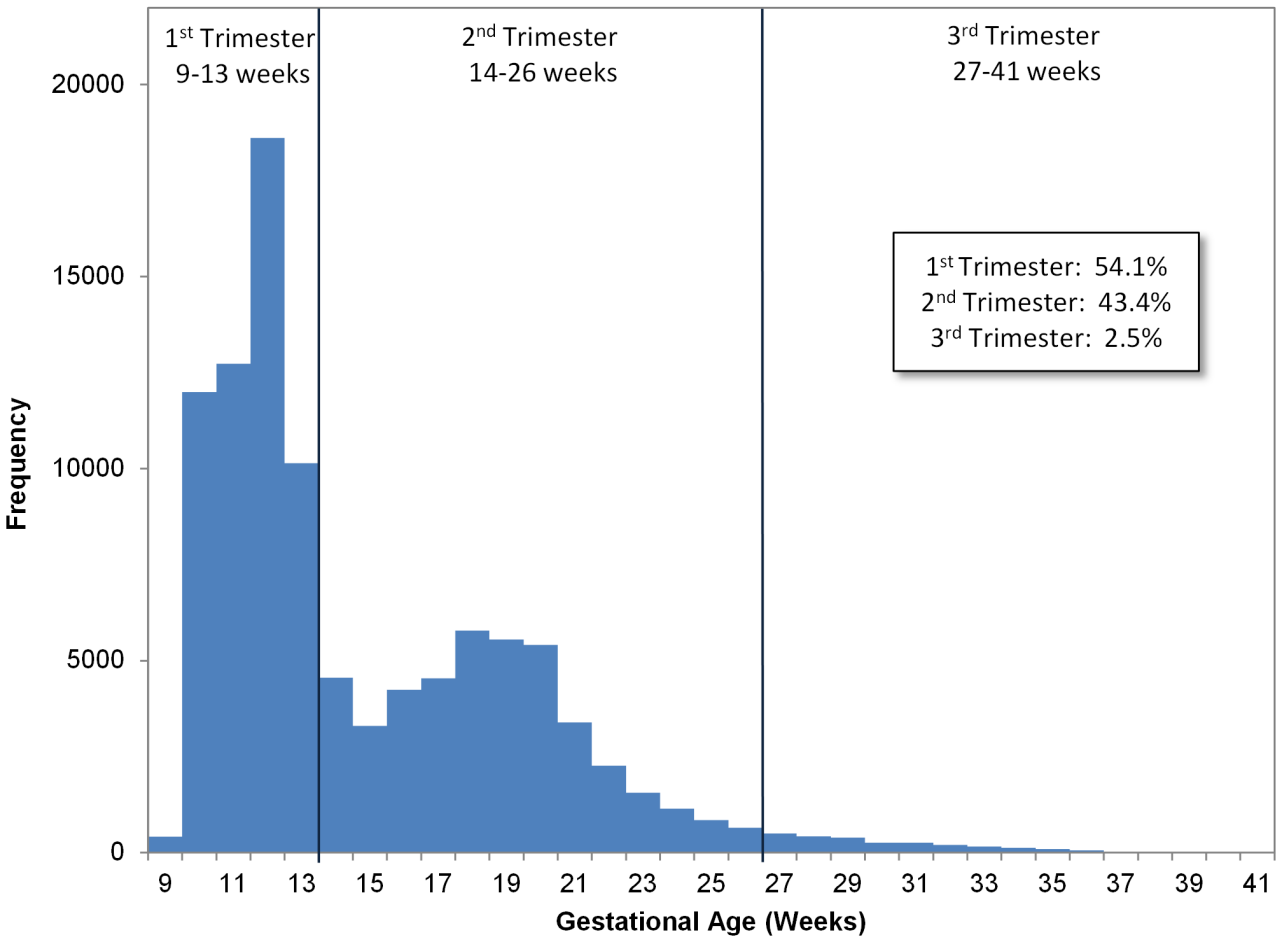
Gestational Age Distribution at Time of Non-invasive Prenatal Testing. This figure shows the frequency distribution by week of the gestational age of the fetus at the time testing. Percent of patients in each trimester is displayed in the inset table.

The average NIPT patient underwent testing at 15 weeks, 3 days gestation, was 35.1 years of age at the time of testing, and had a BMI of 27.1 kg/m^2^. Of those tested, 3.5% carried a multifetal gestation (Table 1). The distribution of gestational age at the time of NIPT is bimodal (Figure 1) with 54.1% of the patients tested in the first trimester, 43.4% in the second trimester, and the remaining 2.5% in the third. The gestational age distribution for the first population is tightly centered around 12 weeks and the second is centered between 18–19 weeks. This bimodal distribution is a result of the difference in clinical care based on the indications for testing including advanced maternal age (≥35 years of age at the due date in singleton pregnancies; ≥32 years of age at term in twin pregnancies), personal or family history, ultrasound findings, and positive serum biochemistry screening test results. Because both maternal age and history are known prior to clinical findings, testing can begin as early as 10 weeks' gestation (the first distribution). At the second distribution, 16–20 weeks' gestation, women who otherwise would be low risk may begin to present with clinical findings including abnormal ultrasound findings and positive serum biochemical screening test results, indicating that further – usually invasive diagnostic - testing is recommended.

**Table 1:** Average NIPT Patient Demographics (n = 100,000).

Average NIPT Patient Demographics (n = 100,000)	
Maternal Age	35.1 years
Gestational Age	15 wks, 3 days
Multifetal Gestations	3,530 (3.5%)
Maternal Weight	72.82 kg
Maternal Height	1.63 m
Maternal BMI	27.1 kg/m^2^

Gestational age was determined by LMP or ultrasound. Maternal height and weight are not required for testing and not provided for all samples, n = 86,734.

**Figure 2:**
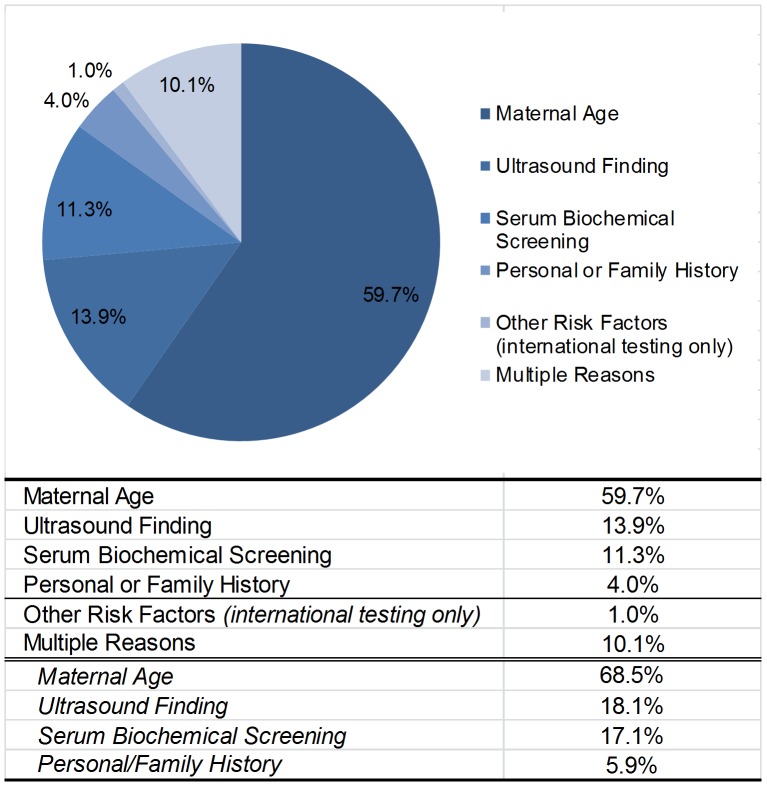
Clinical Reasons for Non-invasive Prenatal Testing. This figure shows the clinical indication for testing. Advanced maternal age is defined as maternal age at birth of 35 or greater for singletons, 32 or greater for twins and 27 or greater for triplets or more. Subtotals for multiple indications include any time the indication is selected.

The largest indication for further testing is advanced maternal age at 59.7%. This is followed by ultrasound findings, positive serum screening, multiple indications and personal or family history at 13.9, 11.3, 10.1, and 4.0% respectively (Figure 2). The breakdown of the samples with multiple indications for testing is similar to the single indication population with maternal age as the largest subgroup. As expected, the average maternal age of those with an advanced maternal age indication, defined as maternal age 35 or greater for singletons, 32 or greater for twins and 27 or greater for triplets or more, is 37.8, while all other indications for testing average between 29 and 32 years of age.

Operational laboratory performance is primarily measured in turnaround time, defined as the time between the arrival of the sample into the lab to the reporting of the final results by the Laboratory Director. For the 100,000 samples in this study, average the turnaround time was 6.9 calendar days. Only 1.6% of those samples required more than 2 weeks to result (Table 2).

**Table 2:** NIPT Laboratory Performance.

Laboratory Performance Metrics (*n = 100,000*)	
**Turn-around Time for Results**	
Calendar Days	6.97 days
Business Days	4.54 days
>10 Business Days	1.6% (1564)
**Not Reportable Samples**	
Quantity Not Sufficient	0.9% (842)
*Redraws Received*	67.2% (571)
*Success Rate*	72.9% (416)
Not Reportable - Other	1.0% (1086)
*Redraws Received*	47.9% (493)
*Success Rate*	89.9% (443)
Not Reportable after Redraw	1.3% (1330)
**Canceled or Amended**	
Canceled Tests	0.9% (870)
Amended Reports	0.7% (695)
**Tube Type of Submitted Specimen**	
EDTA	3.3% (3350)
BCT-Cell Free	96.7% (96650)

The table shows key laboratory performance indicators. Business days are defined as Monday through Friday excluding federal holidays. Canceled tests are samples that are inappropriate for testing primarily those with no indication for testing. Amended reports primarily include reports amended for typographical errors.

**Figure 3:**
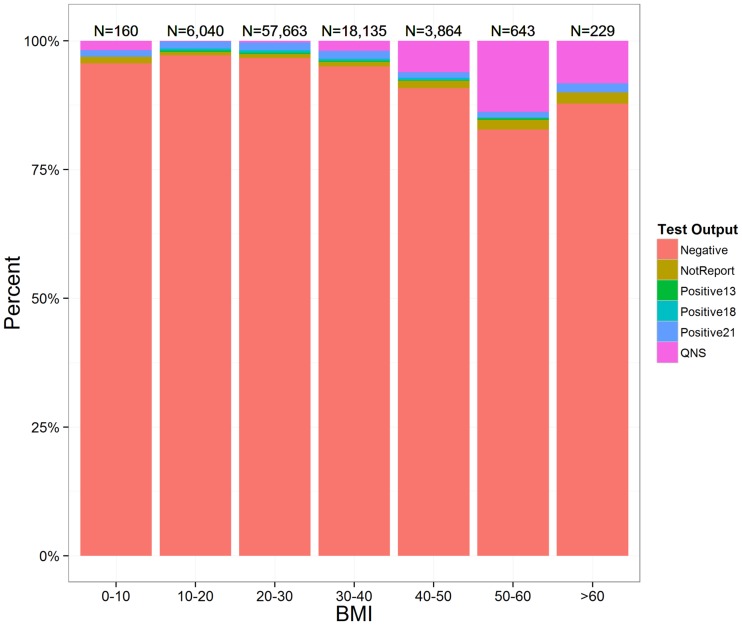
Impact of BMI on Final Results. This figure shows a breakdown of final results when binned by maternal BMI. The number of patients in each bin is displayed above their respective bin.

A secondary measure of clinical performance is the overall non-reportable rate – the percent of samples that do not produce a clinically actionable result. The total Non-Reportable rate was subdivided between insufficient fetal DNA (Quantity Not Sufficient; QNS) and other not reportable etiologies (ONR). QNS samples were samples that contained too little fetal DNA to produce a valid result (less than 4.0% ccffDNA or less than 100 copies of ccffDNA) and represented 0.9% of samples. The laboratory received redraws for 67.2% of the QNS samples with 72.9% success rate in generating a result for the redraw. This reduced the overall QNS rate to 0.54% in all patients. A review of QNS samples does indicate that high BMI increases the chance of a QNS result (Figure 3) with 18.3% of patients with BMI >60 kg/m^2^ resulted as QNS. It is worth noting that severely obese patients >60 BMI have few testing options and NIPT testing was able to provide a clinical result for 79% of these patients. Other non-reportable samples are samples in which the laboratory quality control criteria are not met. This represented 1.0% of all samples. The laboratory received redraws for 47.9% of the NR samples with an 89.9% success rate in generating a result for the redraw. This reduced the overall other non-reportable rate to 0.1% in these patients. Factors that contributed to the NR failures were generally technical or mechanical in nature.

For NIPT, 98.1% of all samples resulted in an unequivocal positive or negative result. The turnaround time for 98.4% of all samples was less than 2 weeks. Sample redraws were received on average 17.6 days from the first draw and rescued an additional 0.6% of samples for an overall result success rate of 98.7%.

### Result Concordance

The overall positivity rate of Trisomy 21 in all high risk pregnancies was 1.51% (a frequency of 1/66) followed by a 0.45% and 0.21% rate for trisomies 18 and 13, respectively (Table 3). Further analysis of the NIPT positivity rates by indication for testing and multifetal status indicates that personal or family history had the lowest overall autosome aneuploidy rate of 0.32% (a frequency of 1/313) and samples with abnormal ultrasound findings the highest with a 3.04% autosome aneuploidy rate (a frequency of 1/33). Of the 11,300 samples identified as high-risk due to positive biochemical serum screening results, NIPT indicates an autosomal aneuploidy positivity rate of only 2.3% (a frequency of 1/258), indicating a 96.0% false positive rate for serum biochemical screening. Previous large clinical studies of aneuploidy rates in women of maternal age ≥35 via amniocentesis show very similar positivity rates to those of NIPT (Table 4). The NIPT positivity rates we have seen in our 100,000 sample experience mirror those predicted by large amniocentesis studies. This is a very reassuring independent validation of test performance [10], [11].

**Table 3:** Breakdown of the NIPT Final Results.

			Increasing Positivity Rate ------>				
	All Samples	Frequency	Personal/Family History Only	Maternal Age Only	Serum Screening Only	Multifetal Gestation	Ultrasound Finding Only
Results	(n = 100,000)	1/X	(*n = *4,038)	(*n = *59,669)	(*n = *11,295)	(*n = *3,530)	(*n = *13,915)
Negative	**95.95%**	**-**	97.5%	96.0%	96.0%	93.9%	95.3%
Trisomy 21	**1.51%**	**66**	0.25%	0.99%	1.76%	1.79%	1.78%
Trisomy 18	**0.45%**	**222**	0.07%	0.27%	0.31%	0.60%	0.78%
Trisomy 13	**0.21%**	**476**	0.00%	0.12%	0.21%	0.23%	0.47%
Not Reportable - Other	**0.84%**	**119**	0.84%	0.82%	0.88%	1.02%	0.92%
Not Reportable - QNS	**1.08%**	**93**	1.31%	1.21%	0.81%	2.44%	0.73%

Trisomy 13 and 18 began reporting in February 2012.

**Table 4:** Study Comparison of Positivity Rate in Advanced Maternal Age Pregnancies.

	*Positivity Rate for Advanced Maternal Age*		
	*NIPT*	*Amniocentesis*	
	McCullough et al.	Forabosco et al.	Ferguson-Smith and Yates
	*Present Study*	*2009*	*1994*
	(n = 59,669)	(n = 51,758)	(n = 52,965)
Autosomes			
Trisomy 21	0.99% (987)	1.00% (517)	1.16% (613)
Trisomy 18	0.27% (266)	0.22% (114)	0.23% (121)
Trisomy 13	0.12% (122)	0.07% (36)	0.07% (39)

Trisomy 13 and 18 began reporting in February 2012.

Accuracy of test results reported by CLIA laboratories is a very important metric to track, however complete individual outcome data is often difficult to obtain. To assess accuracy, we have compiled all of the *ad hoc* feedback we have received regarding false negative and false positive test results from ordering clinicians thru 9 July 2013 (based on karyotype and/or live birth), and compared this to the number of false negatives and false positives we would expect to see based on the performance characteristics established in our clinical validation study (Table 5). As expected, the majority of the outcome data we have received are notifications of false positives and false negatives. Confirmed results are less commonly voluntarily communicated to the laboratory. In total, we were informed of 67 outcomes, 37 of which were false negatives or false positives.

**Table 5:** Clinical Performance Based on Clinical Experience.

	Based On Clinical Validation			
Result	Sensitivity	Specificity	Expected False Positives	Expected False Negatives
T21	100–96.52	99.91–99.42	79 (4–497)	20 (0–341)
T18	100–87.99	100–99.61	0 (0–341)	0 (0–228)
T13	100–51.68	99.92–99.49	79 (4–320)	5 (0–483)

	Based on Clinical Experience			
	Estimated	Estimated	Sensitivity	Specificity
	False Positives	False Negatives	(CI 95%)	(CI 95%)
All				
T21	4	6	99.6%	99.9%
			*(97.3*–*99.9)*	*(99.8*–*100.0)*
T18	5	4	99%	99.9%
			*(93.3*–*100.0)*	*(99.3*–*99.9)*
T13	13	2	98.9%	99.9%
			*(64.6*–*98.5)*	*(99.5*–*99.9)*
Multiple Gestation				
T21	1	0	>99.9%	99.9%
T18	0	1	95.20%	>99.9%
T13	1	0	>99.9%	99.9%

Clinical validation expectation was based on the published validation study. Clinical experience estimates were based on clinical feedback of confirmed discordant results during the duration of the 100,000 testing.

Based on the feedback we have received, the estimated performance of the MaterniT21 PLUS test is within the predicted confidence intervals in the clinical validation studies of both Palomaki, *et al*. and Jenson, *et al*. The sensitivities and specificities were calculated using standard formulas, under the assumption that if the lab was not contacted by the clinician, then the results were not discordant. Though the certainty of the outcome information for all 100,000 clinical cases is a limitation of the report, the information does provide a general sense of performance for the clinical community. Another laboratory offering NIPT for fetal aneuploidy has published data on their clinical experience to date. Futch *et al.* described the clinical experience based on 6123 samples tested using a low coverage whole genome sequencing methodology as well [22]. The performance from that population also reflected their published clinical studies [4].

**Figure 4:**
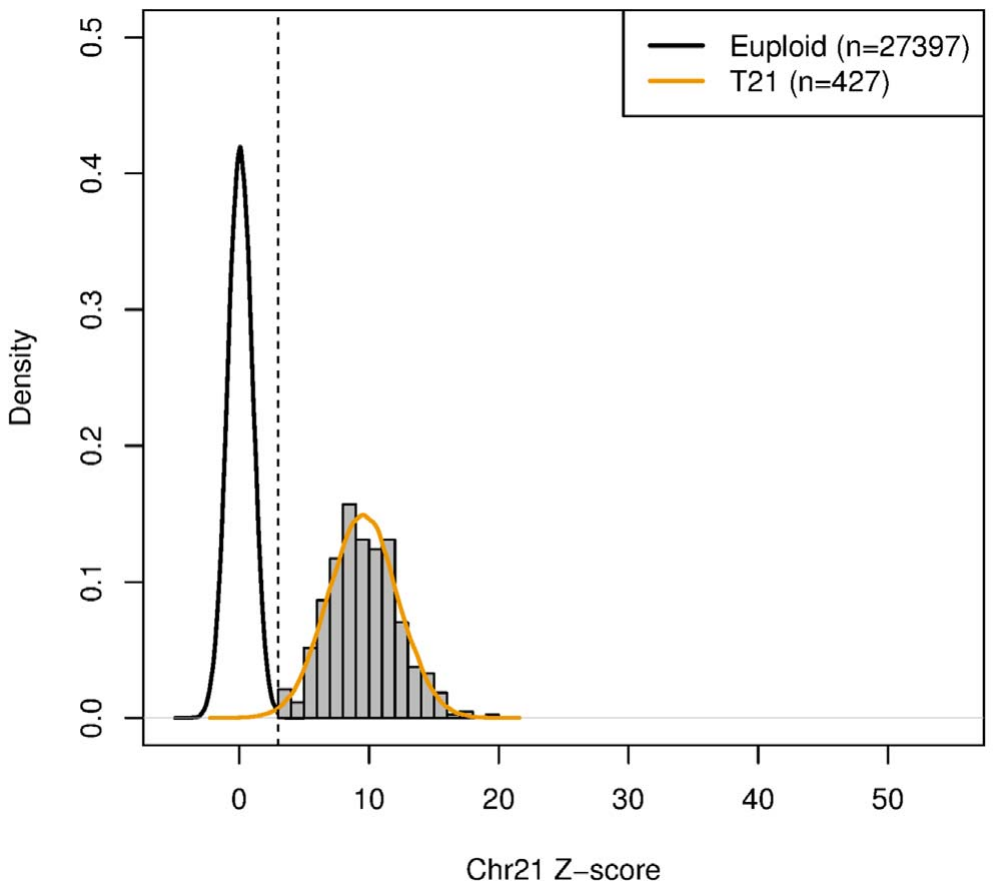
NIPT T21 Modeled Performance at Low Fetal Fractions. In figure, 27,824 samples that passed all laboratory quality criteria with fetal fractions between 4 and 8% were fitted into two normal distributions, one for euploids and one for T21 positives. The fitted distribution was used to estimate specificity and sensitivity.

In addition to using clinical feedback to assess the test performance we have also used the clinical data to model the performance of the test at various fetal fractions. Using a fitted distribution on the resulted trisomy 21 samples and euploids we were able to estimate the sensitivity and specificity as illustrated in Figure 4. As shown, performance within the lower fetal fraction population is not significantly compromised relative to overall published clinical performances, nor is there is significant increase in non–reportable samples. This is fundamentally due to the lack of a “grey zone” for aneuploidy classification. Similar conclusions within the 4–8% fetal fraction zone for trisomy 18 & 13 can be drawn (data not shown). Both clinical feedback and models based on clinical data indicate that the performance of the clinical NIPT is within the performance indicated in the clinical validation studies.

## Discussion

**Figure 5:**
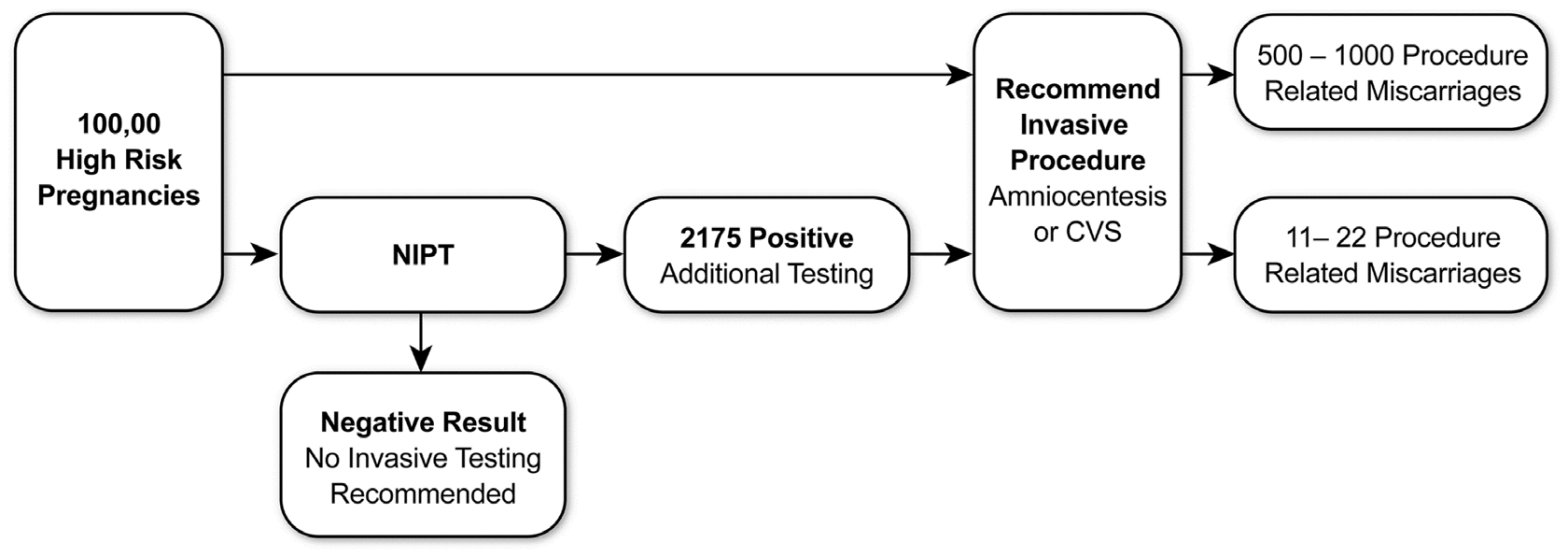
Clinical Impact of Non-invasive Prenatal Testing. All positive NIPT samples are recommended for invasive testing. Invasive procedure related miscarriages numbers based on a 0.5–1.0% frequency range.

The clinical impact of NIPT has been significant as indicated by its quick adoption as a valuable new component within the standards of prenatal care by many clinical institutions. Comparing the data from this NIPT experience with the current clinical paradigm shows the degree of clinical impact. All of the 100,000 women opting for NIPT were high-risk pregnancies and would previously have been counseled, in accordance with ACOG and SMFM guidelines, to consider invasive diagnostic testing. With invasive testing comes risk of procedure-related miscarriages at 0.5–1.0% [12], [14], [15]. This results in the potential for an additional 500–1000 miscarriages if all 100,000 women undergo invasive testing as suggested by the current clinical paradigm. With upfront non-invasive testing, only 2175 of these 100,000 women would continue to be considered high-risk; thus, NIPT reduces the potential need for invasive diagnostic testing significantly, along with the attendant procedure-related miscarriages (Figure 5). For multifetal pregnancies, the expected overall rate of pregnancy loss in twin pregnancies following an invasive procedure is reported to be 3.2% for CVS and 2.9% for amniocentesis. The risk of losing at least one fetus is more than tripled in amniocentesis at 9.3% [13]. In this study of 3519 multifetal gestations, only 92 were identified as having a positive NIPT result. Thus, there exists a potentially significant reduction in the number of patients who are ultimately candidates for invasive testing, with a concomitant reduction in the attendant risk of fetal loss.

In addition to significantly decreasing the need and number of invasive procedures, NIPT has given us novel insights into clinical results which would not have been detected by CVS or amniocentesis. In one example, a result demonstrated unusual data in all three autosomal chromosomes, each one exhibiting a very strong signal indicating an overrepresentation or underrepresentation. The results were reproducible signifying that this was a sample effect and not a technical issue. The patient was subsequently diagnosed with metastatic carcinoma, where we would expect the cell free DNA to have a representative component from the tumor, explaining why the algorithms detected severe deviations in 3 different chromosomal representations. Repeat testing post-surgical and chemotherapeutic intervention showed normalization of all z-scores. In a second case, we again saw irregularities in all three chromosomal z-scores and, in discussing with the clinician, were informed that the patient had large uterine fibroids. These fibroids were subsequently removed and karyotyped, which showed chromosome gains and losses mirroring our NIPT findings. Both cases demonstrate scenarios where a neoplasm may contribute additional cell free DNA to the maternal plasma and confound the aneuploidy results.

The implementation of massively parallel sequencing in assessing for fetal aneuploidy in a non-invasive fashion is a powerful tool. The rapid adoption by the clinicians and their patients has been impressive, and the quick acceptance and support from the professional societies, as well as many payers, clearly shows that incorporating NIPT in the management of pregnancies at high risk for aneuploidies has become an integral part of the standard of care. The additional information provided by NIPT gives a patient and her clinician a better vantage point from which to decide whether or not an invasive procedure is necessary. How NIPT may become part of the routine management of average risk pregnancies remains to be seen, but many, including this group, are assessing different models of how that might evolve.

The MaterniT21 PLUS assay has now been in clinical use for more than two years. Over that time, we have seen an evolution of the test not only in content, but also in the performance of the bioinformatics analysis. Positivity rates have been consistent month to month, and have been at levels predicted by previous large amniocentesis studies that evaluated similar populations. A close assessment of the clinical feedback that we have received on 100,000 cases tested using the latest version of our bioinformatics workflow strongly suggests that the assay is estimated to be performing at or better than the performance characteristics established in the original clinical validation study (Palomaki, *et al*.; Jensen, *et al*.). There are known biological reasons for discrepant NIPT results, and we have come across examples of each of them in the clinical laboratory, which explains some of the false positive and false negatives we have seen in the lab. Because the source of fetal cell free DNA is the placenta, the test is akin to a liquid CVS. As a result, confined placental mosaicism (CPM) may account for a portion of the false positives. Additionally, an early fetal demise, noticed or unnoticed, may also result in false positive result. False negative results may arise from “reverse” CPM, where while the fetus is determined to be trisomic, the placental cell line may have undergone a trisomy rescue event where the predominant cell line of the placenta is euploid. Postpartum placental studies have been performed in some of our discrepant cases demonstrating each of these biological phenomena. Additionally, any confounding factor, such as an unknown chromosomal abnormality in the maternal cell line (mosaic or otherwise), may result in an inaccurate result. Finally, as with any test that has a human component to it, lab error may also occur.

Several different laboratories have developed varying approaches to noninvasive testing for fetal aneuploidy, for a comprehensive review of NIPT see Norwitz *et al.* [23] and have demonstrated strong clinical accuracy, though a detailed look reveals clear differences among the various assay methodologies.

The first difference is a directed vs. whole genome approach to sequencing. While there is an advantage in throughput by using a directed approach that assesses limited portions of the genome, the content is locked to these regions of interest, which does not allow for assessing chromosome abnormalities outside of the targeted regions. Moreover, if a directed approach were to be expanded to include additional content, there would be a sacrifice of throughput capabilities. A whole genome approach, while having a somewhat reduced throughput, inherently has the potential to analyze regions throughout the genome since the data is already being captured, making it primed for incorporating additional content such as microdeletion/microduplication syndromes and single gene disorders (β-thalassemia) [19]–[21].

A second distinguishing factor is the bioinformatics algorithm (“pipeline”) itself and the utility of the result that comes out of it. Generating next generation sequencing data has become fairly straightforward. Nonetheless, it remains critical that the data is analyzed and interpreted properly. Both directed and whole genome approaches have shown that there is a general separation between euploid and aneuploid samples in their analyses and, for the most part, a case clearly falls into one category (Negative), or the other (Positive), making the reporting of such a case straightforward [1], [2], [4], [5], [16]. The challenge lies in how to assess and report out the cases that hover between these two distinct groupings. This occurs routinely with partial duplications, mosaicism, or samples with a lower than average fetal fraction. The ability to deal effectively with these challenging cases, though less common, is an important distinguishing factor between the different NIPT assays.

In the study by Bianchi, *et al*. [4], a “grey zone” was used to address all samples in this borderline, challenging range. Cases that had a normalized chromosome value within 2.5–4.0 were deemed “unclassified”, and a determination of ploidy status could not be made in 14 out of 499 cases (2.8%). As a result, all patients with these results did not receive clinically actionable NIPT results and they were defaulted to the original invasive testing algorithm. Combined with the cases that had an insufficient fetal fraction for testing by their methods, this amounted to a total non-reportable rate of 5.8%. Similarly, the assay described by Zimmerman, e*t al*. [16] seem required a higher fetal fraction in order to routinely provide an interpretation. The cases that failed their quality requirements included 90% of cases having a fetal fraction between 4.0 and 6.0 percent, and 16.7% of cases having a fetal fraction between 6.0 and 8.0 percent, and these cases did not receive a clinically actionable result. Thus, the total non-reportable rate was 12.7%. The MaterniT21 PLUS assay was designed with the goal of establishing an analysis pipeline and laboratory process that is sensitive enough to provide clear cut, actionable results. To that end, a straightforward threshold for positivity, without a borderline, suspicious, suspected, or inconclusive parameter obviated the need for a grey zone. Every sample that meets quality criteria (>98%) is resulted. The MaterniT21 PLUS assay was designed to be highly sensitive, even at lower fetal fractions. The performance characteristics were established in samples with a minimum fetal fraction of 4%, and the performance has been confirmed by our extensive clinical experience in the same clinical popluation.

In addition to providing a novel way of assessing aneuploidy risk in pregnancy, NIPT has given us unprecedented access to the placenta and insights to the mother's health. While placental mosaicism may be a confounding factor in any noninvasive assessment of the fetus, it can still provide us with useful information in regards to the possibility of placental insufficiency and intrauterine growth restriction [17], [18].

## Summary

The introduction of NIPT has caused a paradigm shift in how pregnancies are evaluated for the presence of fetal chromosomal abnormalities. Clinicians and their patients now have a much more powerful tool at their disposal to help them make clinical decisions regarding the need for an invasive procedure with its small but significant risk to the pregnancy. The MaterniT21 PLUS assay has shown its reliability in the clinical setting for more than two years, and this review of the laboratory's 100,000 cases further supports the performance of the test. As NIPT technology continues to improve, and content continues to expand, we will all gain greater and greater knowledge about fetuses prenatally.
